# OKseqHMM: a genome-wide replication fork directionality analysis toolkit

**DOI:** 10.1093/nar/gkac1239

**Published:** 2023-01-11

**Authors:** Yaqun Liu, Xia Wu, Yves d’Aubenton-Carafa, Claude Thermes, Chun-Long Chen

**Affiliations:** Institut Curie, Université PSL, Sorbonne Université, CNRS UMR3244, Dynamics of Genetic Information, 75005 Paris, France; Institut Curie, Université PSL, Sorbonne Université, CNRS UMR3244, Dynamics of Genetic Information, 75005 Paris, France; Université Paris-Saclay, CEA, CNRS, Institute for Integrative Biology of the Cell (I2BC), 91198 Gif-sur-Yvette, France; Université Paris-Saclay, CEA, CNRS, Institute for Integrative Biology of the Cell (I2BC), 91198 Gif-sur-Yvette, France; Institut Curie, Université PSL, Sorbonne Université, CNRS UMR3244, Dynamics of Genetic Information, 75005 Paris, France

## Abstract

During each cell division, tens of thousands of DNA replication origins are co-ordinately activated to ensure the complete duplication of the human genome. However, replication fork progression can be challenged by many factors, including co-directional and head-on transcription-replication conflicts (TRC). Head-on TRCs are more dangerous for genome integrity. To study the direction of replication fork movement and TRCs, we developed a bioinformatics toolkit called OKseqHMM (https://github.com/CL-CHEN-Lab/OK-Seq, https://doi.org/10.5281/zenodo.7428883). Then, we used OKseqHMM to analyse a large number of datasets obtained by Okazaki fragment sequencing to directly measure the genome-wide replication fork directionality (RFD) and to accurately predict replication initiation and termination at a fine resolution in organisms including yeast, mouse and human. We also successfully applied our analysis to other genome-wide sequencing techniques that also contain RFD information (e.g. eSPAN, TrAEL-seq). Our toolkit can be used to predict replication initiation and fork progression direction genome-wide in a wide range of cell models and growth conditions. Comparing the replication and transcription directions allows identifying loci at risk of TRCs, particularly head-on TRCs, and investigating their role in genome instability by checking DNA damage data, which is of prime importance for human health.

## INTRODUCTION

The faithful transmission of genetic information to daughter cells is crucial for maintaining genome stability. In humans, at each cell division, tens of thousands of replication origins need to be co-ordinately activated to ensure the complete duplication of the >6 billion base pairs (bp) of the human genome. However, cells are routinely exposed to endogenous and exogenous stresses that might alter the DNA replication program, increasing the risk of some diseases. For instance, replication stress-induced genome alterations can represent an important early cause of cancer (1).

The progression of replication forks can be challenged by many factors, including transcription-replication conflicts (TRC) due to the fact that the replication and transcription machineries share the same DNA template. TRCs can be co-directional or head-on, and the latter has been shown to be more dangerous for genome integrity (2). Previous bioinformatic analyses revealed that in many different species, from bacteria (3) to humans (4,5), the transcription of most genes is co-directional with the replication forks to avoid head-on TRCs. In 2016, Petryk *et al.* showed that replication fork directionality (RFD) can be directly measured genome-wide by sequencing Okazaki fragments (OK-seq) (6), which are present only on the lagging replicating strand. This method allows the quantitative analysis and the accurate detection of replication initiation and termination zones. The analysis of OK-seq data from human cells showed a significant co-directionality of replication fork progression and transcription of active genes (6).

Other techniques also provide genome-wide RFD information, such as polymerase (Pol) usage sequencing (Pu-seq) (7), enrichment and Sequencing Protein-Associated Nascent DNA (eSPAN) (8), Sister Chromatids After Replication by DNA sequencing (SCAR-seq) (9), Genome-wide Ligation of 3′- hydrOxy Ends sequencing (GLOE-seq) (10), and Transferase-Activated End Ligation sequencing (TrAEL-seq) (11). Pu-seq identifies embedded ribonucleotides in *Schizosaccharomyces pombe* polymerase mutants to determine the distribution of Pol ϵ (that replicates the leading strand) and Pol δ (that replicates the lagging strand), thus measuring the proportion of fork movement in the genome (7). The eSPAN (8) and SCAR-seq (9) methods map strand-specific proteins and/or histone modification deposition on replicating DNA strands. Therefore, the obtained genome-wide data on the association of proteins (or histone modifications) specifically with the leading (or lagging) strand can be used also to determine the replication fork directionality. GLOE-seq (10) and TrAEL-seq (11) were originally developed to map 3′ ends of single-strand DNA and/or resected double-strand DNA, while they can also indirectly reveal the fork directionality based on these structures associated with canonical and/or reversed replication forks.

Moreover, in recent years, strong evidence has shown that replication- and transcription-related mutational strand asymmetries are common in cancer (12). For instance, APOBEC-associated mutations (also called APOBEC mutation signatures) have been detected in up to 15% of all sequenced human tumour samples and contribute to 50% of all mutations in many tumours. APOBEC-associated mutations preferentially occur on the lagging-strand template during DNA replication, and are strongly associated with mismatch repair and transcription-coupled damage repair in cancer (13–17). Furthermore, N6-methyladenosine modifications are among the most prevalent internal modifications in mammalian mRNAs and are implicated in both physiological and pathological processes. Importantly, aberrant regulation/expression of N6-methyladenosine and of its modulators (e.g. methyltransferase-like 3) is a common feature in various tumour types (18–20). It has been shown that methyltransferase-like 3 and N6-methyladenosine could promote homologous recombination-mediated repair of double-strand breaks by modulating DNA-RNA hybrid (R-loop) accumulation (21). Importantly, we and others have recently demonstrated that R-loops frequently accumulate at transcription termination sites of actively transcribed genes with a high frequency of head-on TRCs (22,23). Therefore, systematically unveiling DNA replication features genome-wide is essential for human health.

**Figure 1.**
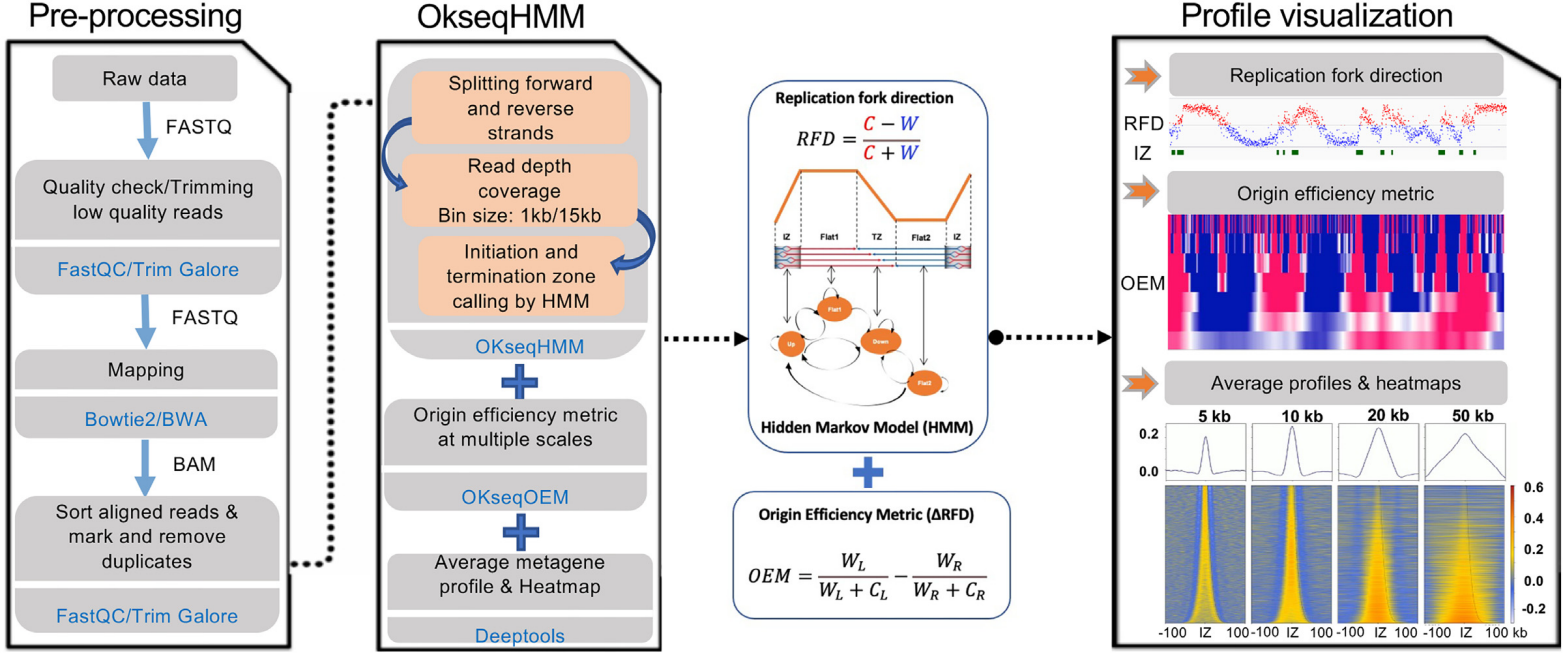
Schematic presentation of the data analysis pipeline in the OKseqHMM toolkit. Raw sequencing data are pre-processed into aligned files with the bioinformatics tools indicated in blue (Pre-processing panel). The middle panel shows the major functions of the OKseqHMM toolkit. The first function (OKseqHMM) checks the input aligned bam files to determine whether they are single- or paired-end sequencing data, and then automatically splits the reads into Watson and Crick strands and computes the replication fork directionality (RFD) (OKseqHMM panel). By default, the calculation is performed within 1 kb adjacent windows (recommended for human cells) and then smoothed into 15 kb sliding windows with 1 kb step. These parameters can be easily adjusted based on the nature of the data. Different replication features, i.e. initiation zones (IZ), two intermediate states and termination zones (TZ), are predicted using an HMM algorithm (see the graphic presentation of the four HMM states and their dynamic transition scheme). The second function (OKseqOEM) uses the reads on Watson and Crick strands to generate origin efficiency metrics (OEM) at multiple scales to visualize the RFD transition. The last function allows users to generate an average metagene profile and heatmap to analyse the RFD and OEM distributions around the genes/regions of interest. Results can be visualized in genomic visualization browsers (such as IGV), as shown in the right panel.

However, to date, no bioinformatic tool has been developed to analyse RFD data and to determine the replication initiation and termination positions genome-wide, although several methods have been previously described for OK-seq data analysis, for instance, the Hidden Markov Model (HMM) to analyse human OK-seq data (6) and origin efficiency metric (OEM) for yeast OK-seq data (24,25). Therefore, to set up a uniform framework for OK-seq data (and related data) analysis, we developed an integrative bioinformatics toolkit, called OKseqHMM, to directly obtain RFD profiles genome-wide and at high resolution. In addition to the fork progression direction, this toolkit also gives information on replication initiation/termination zones and on long-travelling unidirectional forks using an algorithm based on HMM, and calculates the OEM to visualize the transition of RFD profile at multiple scales. It then generates the average metagene profiles and heatmaps to visualize the RFD/OEM distribution along the regions of interest (Figure 1). Using OKseqHMM and a large number of publicly available OK-seq datasets (13 in total) from *S. cerevisiae*, mouse and human cells, we successfully obtained high-resolution (∼1 kb for mouse and human cells and ∼50 bp for yeast) RFD profiles and accurate calling of the corresponding replication initiation and termination zones genome-wide.

## MATERIALS AND METHODS

The OKseqHMM toolkit is an R package for profiling OK-seq data to study the genome-wide replication program. This multi-function toolkit accepts OK-seq data from the original mapping bam file(s) for matrix counting, RFD calculation, initiation/termination zone calling, and average metagene profile/heatmap creation.

### The OKseqHMM function measures RFD and predicts replication initiation/termination zones

OKseqHMM is the main function of this toolkit and involves several important OK-seq data analysis steps (Figure 1). The function transforms OK-seq data into RFD profiles for a first visualization (e.g. with genomic visualization browsers, such as IGV). Then, it can accurately identify replication initiation zones (IZs, upward transitions in the RFD profile), termination zones (TZs, downward transitions in the RFD profile), and intermediate states (flat RFD profile) along the genome using an HMM. The OKseqHMM function checks the input (i.e. aligned bam files) to determine whether they are single- or paired-end sequencing data, then automatically splits reads into Watson and Crick strands and calculates the RFD. For paired-end sequencing data, users can select one of the three following modes: (i) using all unique mapped reads, (ii) using only paired-mapped reads, or (iii) using only properly paired-mapped reads.

For each window, RFD was computed as follows:

\begin{equation*}{\boldsymbol{RFD\ }} = \frac{{{\boldsymbol{C}} - {\boldsymbol{W}}}}{{{\boldsymbol{C}} + {\boldsymbol{W}}}}\end{equation*}

where ***C*** and ***W*** are the number of reads mapped to the Crick and Watson strands, respectively. They reveal the proportion of rightward- and leftward- moving forks, respectively, within each window (e.g. 1 kb window was used for OK-seq data of human cells). As the total amount of replication events on both strands should be constant across the genome, the difference between strands was normalized to the total read count to account for variations in read depth due to, for instance, copy number variations, sequence bias. RFD ranges from –1 (100% leftward-moving forks) to +1 (100% rightward-moving forks), and 0 means equal proportions of leftward- and rightward-moving forks. Data obtained from biological replicates produced RFD profiles that strongly correlated with each other: Pearson *R* = 0.92, *P* < 10^−15^ (*t*-test) for HeLa cells and *R* = 0.93, *P* < 10^−15^ for GM06990 cells. Similar correlations were observed between RFD profiles obtained following EdU or EdC labelling (6).

**Figure 2.**
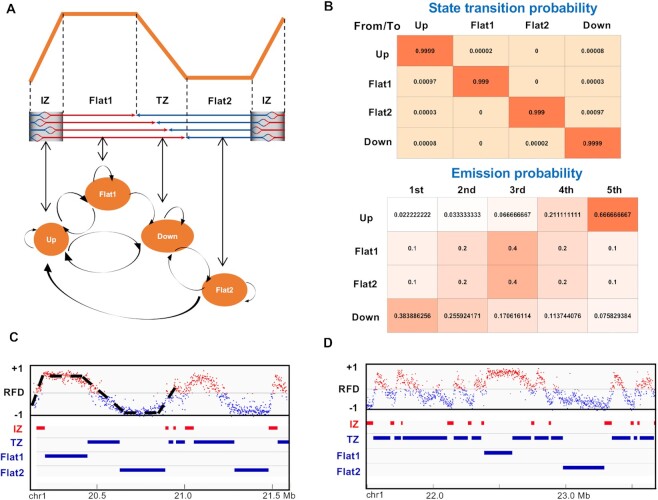
Schematic presentation of the HMM algorithm for initiation and termination zone detection. (**A**) The 4-state HMM model used in the segmentation process: Up, regions of predominant initiation (IZ); Down, regions of predominant termination (TZ); Flat1 and Flat2, two intermediate transition states. (**B**) Default state transition probability (between states) and emission probabilities (probabilities of each state within five quantiles of the $\Delta RFD$ values) used in OKseqHMM (see Materials and Methods for detail). The probability matrixes were colour-coded based on their values (higher probability values are closer to red). (**C** and **D**) Examples of RFD profiles in chromosome 1 of human HeLa cells with the corresponding IZs, TZs and two Flat states identified by OKseqHMM. Each point on the RFD profile gives the RFD value calculated within each 1 kb adjacent window, and the windows with positive and negative RFD values are shown in red and blue, respectively.

A four-state HMM is used in OKseqHMM to detect, within the RFD profiles, the ascending, descending and flat segments that represent regions of predominant initiation (‘Up’ state), predominant termination (‘Down’ state), and constant RFD (‘Flat1’ and ‘Flat2’ states), respectively (6) (Figure 2A). In the HMM segmentation process, the RFD values were computed within 15 kb (for human OK-seq data) sliding windows (by default, stepped by 1 kb across the autosomes). The HMM used the $\Delta RFD$ values between adjacent windows, in which $\Delta \ RF{D}_n = \frac{{RF{D}_{n + 1} - RF{D}_n}}{2}$ for the window *n*. By default, windows with < 30 reads on both strands were masked. The $\Delta RFD$ values (between –1 and 1) were divided into five quantiles. Then, the HMM package of R (http://www.r-project.org/) was used to perform the HMM prediction with probabilities of transition and emission that are manually defined using the training dataset (Figure 2B). The same transition and emission probabilities used in our previous study (6) were set as default values and used in all OK-seq data analyses in the current study. Two representative examples of human RFD profiles with the segments of IZs, TZs and two Flat states obtained by OKseqHMM are shown in Figure 2C, D. The choice of a 15 kb sliding window was based on a compromise between spatial resolution and reproducibility of ascending segment detection among biological replicates. Lastly, the efficiency of the detected ascending segments (i.e. IZs) was estimated as follows:

\begin{equation*}\Delta {\boldsymbol{\ RF}}{{\boldsymbol{D}}}_{{\boldsymbol{segment}}} = \frac{{{\boldsymbol{RF}}{{\boldsymbol{D}}}_{{\boldsymbol{end}}} - {\boldsymbol{RF}}{{\boldsymbol{D}}}_{{\boldsymbol{start}}}}}{2}{\boldsymbol{\ }}\end{equation*}

where ***RFD_start_*** and ***RFD_end_*** correspond to the RFD values computed in 5 kb windows around the left and right extremities of each segment, respectively.

### The OKseqOEM function generates the multi-scale RFD transition profiles

To investigate origin efficiency (i.e. $\Delta RFD$), OkseqHMM toolkit includes a second function (OKseqOEM) to visualize this directly at multiple scales (Figure 1). As defined in the previous publication on yeast OK-seq data analysis (24), the densities of Okazaki fragments on the Watson and Crick strands were compared within four fixed-size sliding bins, which are strand-specific 10 kb quadrant values, to calculate the OEM as follows: $OEM\ = \frac{{{W}_L}}{{{W}_L + {C}_L}}\ - \frac{{{W}_R}}{{{W}_R + {C}_R}}$. *W_L_* and *W_R_* measure the read density in the left and right quadrants on the Watson strand, respectively, while *C_L_* and *C_R_* measure these densities on the Crick strand. Their values range from –1 to 1 for each base in the genome. The highest OEM scores represent replication origins, whereas the lowest scores represent regions of replication termination. In addition, the different amplitudes of positive OEM values (from 0 to 1) describe the origin firing efficiency. Similarly, the degree of termination at each position can be measured from 0 to –1.

Here, OEM calculation was extended within a fixed window size to multiple scales to better fit OK-seq data analysis to other organisms, such as human cells:

\begin{equation*}{\mathrm{\ }}{\boldsymbol{OE}}{{\boldsymbol{M}}}_{{\boldsymbol{i\ for\ list}}\left[ {\boldsymbol{n}} \right]} = \frac{{\left( {{{\boldsymbol{W}}}_{{\boldsymbol{i}} + {\boldsymbol{list}}\left[ {\boldsymbol{n}} \right]} - {{\boldsymbol{W}}}_{\boldsymbol{i}}} \right)}}{{\left( {{{\boldsymbol{W}}}_{{\boldsymbol{i}} + {\boldsymbol{list}}\left[ {\boldsymbol{n}} \right]} - {{\boldsymbol{W}}}_{\boldsymbol{i}}} \right) + \left( {{{\boldsymbol{C}}}_{{\boldsymbol{i}} + {\boldsymbol{list}}\left[ {\boldsymbol{n}} \right]} - {{\boldsymbol{C}}}_{\boldsymbol{i}}} \right)}}{\boldsymbol{\ }}\end{equation*}

where ***list[n]*** can be defined by users as a list of windows (e.g. [1, 10, 20, 50, 100]), and ***i*** ranges from 1 to the total length of the data – *list[n]*; ***C*** and ***W*** correspond to the number of reads mapped on the Crick and Watson strands, respectively, within the corresponding windows.

Using the two bam files of reads within the Watson and Crick strands generated by the OKseqHMM function and the annotation coordinates, the OKseqOEM function can automatically calculate the OEM profiles at different defined scales (e.g. from 1 kb to 1 Mb for human cells). This allows directly visualizing the replication transition states and also validating the IZs identified by OKseqHMM and then double-checking the IZ size and boundary.

### The average metagene profile/heatmap visualizes the RFD distribution in specific genomic regions

To analyse RFD distributions around and/or among genomic regions of interest (e.g. the identified IZs or annotated genes), an additional module was developed for metadata analysis. Using the gene coordinates (or IZs) and the RFD and/or OEM big wiggle files generated with the OKseqHMM and/or OKseqOEM functions, the corresponding profiles/heatmaps can be easily generated with the computeMatrix and plotProfile/plotHeatmap functions of deepTools (https://deeptools.readthedocs.io/en/develop/index.html) (26) by defining the genomic distances of interest for the upstream and downstream borders (Figure 1).

### HeLa S3 cell OK-seq data generation

HeLa S3 cells were cultured in DMEM high-glucose medium (ThermoFisher) with 10% fetal calf serum (Eurobio Scientific) OK-seq libraries were generated starting from exponentially growing cells as previously described (6,27). Libraries were sequenced on an Illumina NextSeq 500 sequencing system using Paired-end (75 cycles).

## RESULTS

### Genome-wide replication fork directionality and origin detection in yeast

**Figure 3.**
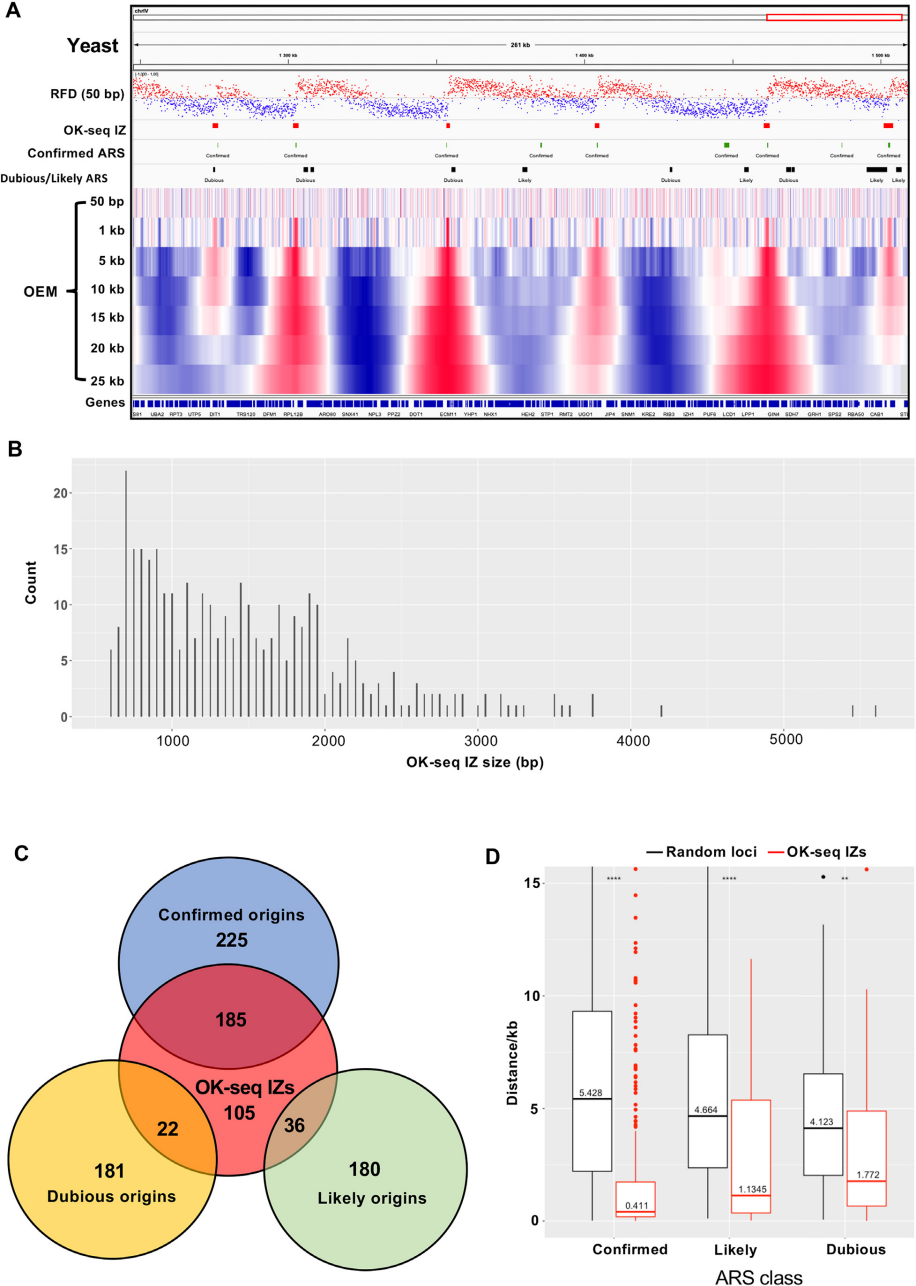
Analysis of yeast OK-seq data by OKseqHMM. (**A**) RFD profile calculated at the 50 bp resolution with the corresponding IZs identified by OKseqHMM that are highly correlated with the confirmed yeast ARS from OriDB (29). The RFD profile is like in Figure 2C, but with a 50 bp resolution (instead of 1 kb). The lower part of the panel shows the OEM profiles calculated from the 50 bp to the 25 kb scale. The windows with positive and negative OEM values are shown in red and blue, respectively. (**B**) Length distribution of the detected OK-seq IZs. (**C**) Venn diagram showing the overlap of OK-seq IZs with all the known yeast origins (ARS) from OriDB clustered in three classes (confirmed, likely, and dubious). Overlap means that the closest distance between the centres of the IZ and ARS is <2 kb. Note that not all confirmed OriDB origins overlapped with OK-seq IZs because all origins might not be active in the culture condition and/or yeast strain used for the OK-seq experiment. Further comparison with origins identified in datasets obtained with other techniques can be found in Figure 7B. (**D**) Boxplot showing the distribution (in red) of distances between the centre of an IZ detected by OKseqHMM and the centre of the closest origin from OriDB (grouped in three classes). Such distances are much smaller than the distances between OriDB origins and the random simulation control (in black), as indicated by the Wilcoxon rank sum test; **P < 10^−2^, ***P < 10^−3^, ****P < 10^−4^.

To evaluate the toolkit performance, first, the available yeast OK-seq data were used (28). OKseqHMM generated the RFD profile at a fine resolution (50 bp), the OEM profiles at different scales (from 50 bp to 25 kb), and a precise IZ/origin calling (Figure 3A). The RFD profiles obtained from the two biological replicates were highly correlated (Pearson *R* = 0.99, *P* < 10^−15^) (Supplementary Figure S1A). About 350 IZs robustly identified by OKseqHMM in both replicates were retained. Their length ranged from 0.5 to 5.5 kb (mean length: 1.5 kb) (Figure 3B, Table 1). To check the accuracy of the IZ calling results, the OK-seq IZs were compared with the known yeast origins [i.e. autonomously replicating sequence (ARS) from OriDB 2.1.0 (29)]. Up to 70% of the detected IZs were at ≤ 2 kb distance (between centres) from a known ARS (Supplementary Figure S1B). As expected, OK-seq IZs correlated better with confirmed ARSs: 185, 36, and 22 IZs overlapped (i.e. distance between centres ≤2 kb) with confirmed (median distance 0.27 kb), likely (median distance 0.48 kb), and dubious (median distance 0.69 kb) origins, respectively (Figure 3C). When all OriDB origins were considered, instead of only the overlapping ones, the distances between OK-seq IZ centres and the closest OriDB origins of each class were still significantly smaller (median distance: 0.41, 1.13 and 1.77 kb for confirmed, likely, and dubious origins, respectively) than when using randomly simulated genomic positions (Figure 3D).

**Table 1.** All OK-seq data analysed by OKseqHMM

Cell line	Cell type/origin	Replicates	Initiation zones		Termination zones		Accession number (reference)
			Number	Size (kb) Mean ± SD	Number	Size (kb) Mean ± SD	
BL79	Burkitt's lymphoma		7798	29 ± 18	7791	211 ± 244	ENA: PRJEB25180 (27)
GM06990	Lymphoblastoid cells	2*	5684	33 ± 19	5715	182 ± 166	SRA: SRP065949 (6)
HeLa MRL2	Epithelial cells from adenocarcinoma	2*	9836	31 ± 18	9441	141 ± 144	SRA: SRP065949 (6)
HeLa S3	Epithelial cells from adenocarcinoma		9089	32 ± 19	9084	223 ± 245	GEO: GSE193547 (Current study)
IARC385	B lymphocytes from Burkitt's lymphoma		4465	36 ± 19	4455	125 ± 164	ENA: PRJEB25180 (27)
IB118	Leiomyosarcoma		3645	26 ± 16	3640	428 ± 440	ENA: PRJEB25180 (27)
IMR90	Fibroblasts		12482	26 ± 17	12468	151 ± 147	ENA: PRJEB25180 (27)
K562	Late-stage chronic myeloid leukaemia		6982	28 ± 15	6967	136 ± 158	ENA: PRJEB25180 (27)
mESC E14	Mouse embryonic stem cells		3370	27 ± 14	3347	483 ± 554	GEO: GSE142996 (8)
Raji	Burkitt's lymphoma		8096	29 ± 16	8080	143 ± 135	ENA: PRJEB25180 (27)
TF1	BCR-ABL negative cell line from erythroblasts		8377	27 ± 17	8371	196 ± 193	ENA: PRJEB25180 (27)
TLSE19	Leiomyosarcoma		10500	27 ± 17	10492	146 ± 144	ENA: PRJEB25180 (27)
Yeast cells	*S. cerevisiae*	2*	348	1.5 ± 0.7	787	14 ± 13	ENA: PRJEB36782 (28)

*If data of biological replicates are available, the profiles obtained with the combined data were used in the figures, and only the segments (i.e. IZs and TZs) reproducibly identified in both biological replicates were retained. SD means Standard Deviation.

Next, the RFD profiles and identified IZs were precisely compared in the two biological replicates to evaluate the performance and reproducibility of the results obtained with OKseqHMM. Although the RFD profiles were very close to each other, the IZs identified in each replicate were not identical (Supplementary Figures S1A and C) due to the local variation in RFD profiles. Amongst the IZs identified in both replicates, ∼60% overlapped with the OriDB confirmed origins (Supplementary Figure S1C), suggesting that they were bona fide origins. Conversely, only a small percentage of IZs detected only in one replicate (called here specific IZs) overlapped with OriDB origins (29% and 40% for replicate 1 and 2, respectively) indicating that they might contain a significant amount of false positives. As expected, IZs that overlapped with OriDB origins displayed a nice RFD transition around their centres, particularly those robustly detected in both replicates (Supplementary Figure S2A). IZs identified in both replicates, but not overlapping with OriDB origins, also presented a strong RFD transition (Supplementary Figure S2B), with negative RFD on the left and positive RFD on the right side, respectively. Conversely, replicate-specific IZs not overlapping with OriDB origins showed a modest level of local RFD transition and a flat RFD profile nearby, again suggesting that they might not be bona fide origins. Lastly, OriDB origins that did not overlap with OK-seq IZs showed almost flat RFD profiles (Supplementary Figure S2C). This suggests that they might not have been active in the yeast cells that were used to generate the OK-seq data because some origins can be growth-condition specific (28). To determine whether an additional parameter could be found to discriminate between good and noisy IZs, firing efficiency (i.e. $\Delta RFD$), size, and probability of confidence of each IZ (obtained from the HMM estimation) were compared (Supplementary Figure S2D–F). As expected, firing efficiency was higher, IZ size was larger, and confidence of detection was higher for IZs that overlapped with confirmed origins and that were identified in both replicates than for replicate-specific IZs non-overlapping with confirmed origins. However, as the distributions of all these three parameters for the IZs associated with confirmed ARSs and the replicate-specific ones not associated with confirmed ARSs overlapped, it was not possible to identify a cut-off to select only good IZs. In conclusion, selecting the IZs that are reproducibly identified in biological replicates will help to improve the specificity, although this will lead to a slight loss of sensitivity.

### Genome-wide detection of replication fork directionality and initiation zones using OK-seq data from different human cell lines

**Figure 4.**
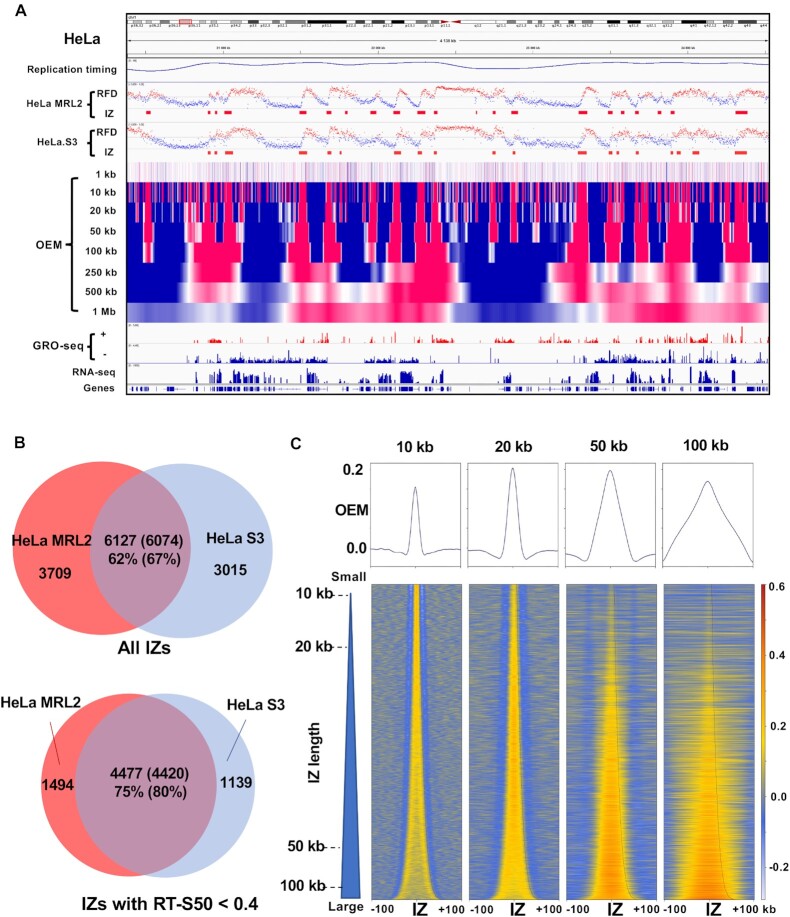
Analysis of HeLa cell OK-seq data by OKseqHMM. (**A**) Replication timing profile obtained by Repli-seq, RFD profiles and the corresponding IZs detected in a publicly available HeLa MRL2 OK-seq dataset (6) and in OK-seq data of HeLa S3 cells generated in the current study, OEM profiles of HeLa S3 cells at the 1 kb to 1 Mb scales, and transcription data provided by GRO-seq and RNA-seq along a ∼4 Mb region on chromosome 1. (**B**) Venn diagrams showing that 67% of OK-seq IZs were shared by the two HeLa cell lines and up to 80% when only the early IZs were considered (i.e. replication timing S50 <0.4). (**C**) Mean OEM profiles and OEM heatmaps (the colour scale is indicated on the right) around the HeLa S3 IZ centres at the indicated scales (10, 20, 50 and 100 kb) sorted by the length of the detected IZs.

Then, the OKseqHMM function was used to analyse previously published OK-seq data from HeLa MRL2 cells (6) and new OK-seq data from HeLa S3 cells, a widely used Encode Tier 2 cell line. The RFD profiles of the two HeLa cell lines were very similar (*R* = 0.86, *P* < 10^−15^), suggesting similar replication programs and IZ positions (Figure 4A). The correlation between HeLa cell lines was slightly lower than the correlation between the HeLa MRL2 cell biological replicates (*R* = 0.92, *P* < 10^−15^) (6), suggesting that the differences between HeLa cell lines were true biological differences and not only technical variations. In each HeLa cell line, ∼10 000 IZs were identified (Table 1), 67% of which were common between cell lines (Figure 4B). IZ conservation was even higher in early-replicating regions: 80% of early IZs were shared between HeLa cell lines (Figure 4B). As expected, these shared IZs showed higher firing efficiency and larger size than IZs specific for each dataset (Supplementary Figure S3A–D). It should be noted that for cell-line specific IZs, the RFD transitions were much stronger in the mean RFD profile of the cell line in which such IZs were detected than in the other cell line. However, the RFD profiles in the other cell line were not completely flat, suggesting that some IZs with low firing efficiency were not detected with our current parameter setting. Interestingly, both shared and cell-specific IZs displayed a high probability of confidence, suggesting a low false positive detection rate (Supplementary Figure S3E).

A very striking difference between human and yeast RFD data was that the size of upward transitions of RFD, therefore the IZ length, ranged between 10 and 50 kb in human cells (∼30 kb on average, which is ∼20-fold larger than the IZ length in yeast), unlike the sharp 1 kb upward transition of RFD at fixed yeast origins (Figure 4A, Table 1). The OEM profile heatmaps, computed around IZs at different scales, showed the strongest positive signals at the corresponding scale: 10 kb scale for small IZs (<10 kb), 20 kb scale for medium IZs (20–50 kb) and 50 or 100 kb for large IZs (>50 kb) (Figure 4C). This confirmed that RFD transitions are associated with the detected IZ length, and also supports the difference between yeast and human OK-seq patterns and the accuracy of IZ detection by OKseqHMM.

**Figure 5.**
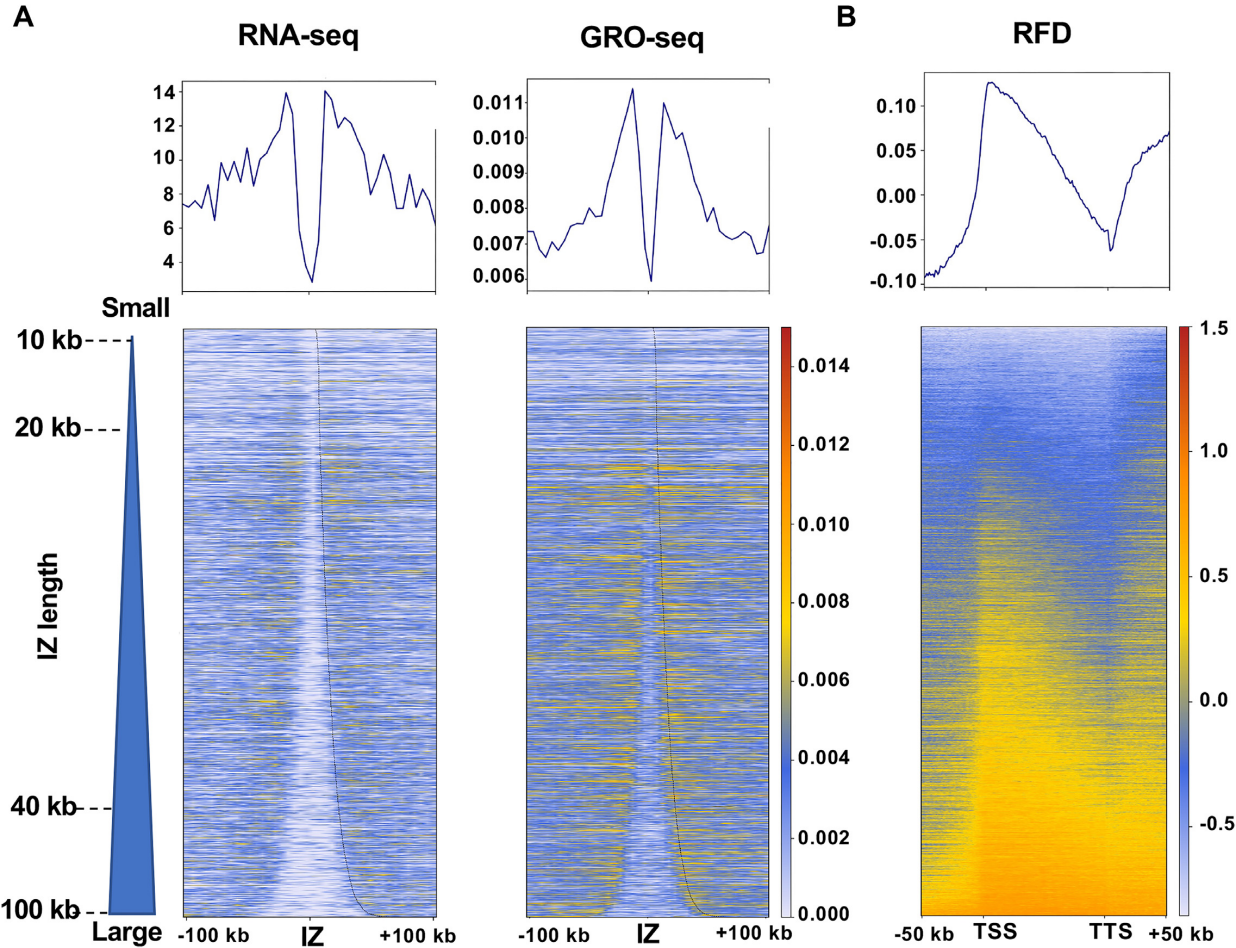
OKseqHMM reveals the coordination between DNA replication and gene transcription. (**A**) Mean profiles and heatmaps of transcription activity (RNA-seq and GRO-seq data) around the HeLa S3 OK-seq IZ centres. (**B**) Mean profile and heatmap of HeLa S3 RFD between the TSS and the TTS of active genes with an extension of ±50 kb. The heatmap colour scales are indicated in each panel.

It has been shown that replication initiation regions are enriched in intergenic regions between active genes (6). To describe how our toolkit can contribute to the analysis of the correlation between DNA replication and gene transcription, the average expression profiles and the corresponding heatmaps (RNA-seq and GRO-seq) were analysed for all detected IZs sorted by length. This confirmed that gene transcription activity was higher in the area immediately surrounding the IZs, and much lower within IZs (Figure 5A). To further compare RFD distribution and gene transcription, the average RFD profiles were calculated as well as the corresponding heatmaps around transcription start sites (TSS) and transcription termination sites (TTS) of 16 336 active genes (RPKM > 1) in HeLa cells with an extension ±50 kb upstream or downstream (Figure 5B). This clearly highlighted frequent replication initiation (upward transition of RFD) in the regions upstream of TSS and downstream of TTS. This leads to a co-directionality between replication and transcription at TSS and a higher risk of head-on TRC at TTS, in agreement with previous publications (6,22). As illustrated also in the study by Promonet et al, combining RFD profiles obtained from OK-seq data with other genomic data (e.g. gene transcription, R-loops, replication fork stalling and DNA damage) allowed us to show that fork pausing at TTS of highly expressed genes containing R-loops prevents head-on TRCs and maintains genome integrity (22).

**Figure 6.**
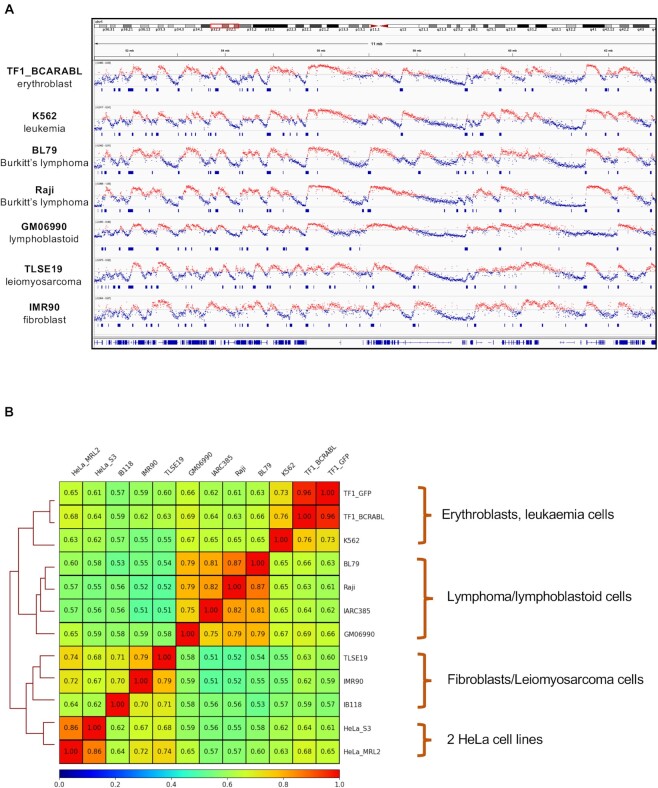
Comparison of the genome-wide RFD profiles of different human cell lines shows the cell type-specific replication program. (**A**) Cell type-specific RFD profiles and the corresponding IZs detected in the indicated human cell lines. (**B**) Pairwise Pearson correlations between OK-seq RFD data (1 kb) for the indicated human cell lines.

In addition to the OK-seq data of HeLa cell lines, the OKseqHMM function was used to reanalyse publicly available OK-seq data from different human cell lines (6,27), such as fibroblasts (IMR90), lymphoblastoid (GM06990) and lymphoma cells (Raji, BL79, IARC385), leiomyosarcoma cells (IB118, TLSE19), leukaemia cells (K562) and erythroblasts (TF1) (Table 1, Figure 6). OKseqHMM generated high-quality cell type-specific RFD profiles and robust IZ calling for all datasets analysed. The average IZ sizes in the different cell types were within the same range (between 26 and 36 kb), demonstrating that it is a common feature of human cells. Conversely, the RFD profiles were cell-type specific, although they were quite conserved among cell lines in some origin-rich regions. The data obtained for similar cell types or for cell lines with a similar origin showed similar RFD profiles (Figure 6B). For instance, the Pearson correlation *R* was 0.87 between the Raji and BL79 cell lines (both derived from lymphoma samples) and 0.79 between these cell lines and GM06990 human lymphoblastoid cells. Similar results were obtained when comparing the RFD transitions (i.e. OEM profiles) and the IZs identified in the different cell types (Supplementary Figure S4A and B). Approximately two-thirds of IZs were shared between close cell types. This percentage decreased to 40–50% for cell types of different origin (Supplementary Figure S4B, bottom left). Comparison for the IZs located within the constant early replicated regions defined in the study by Marchal *et al.* (30) showed that ∼65% of early IZs were common amongst different cell types, and frequently up to 80% between close cell types (Supplementary Figure S4B, top right).

### OKseqHMM to analyse the RFD profiles from other sequencing data

**Figure 7.**
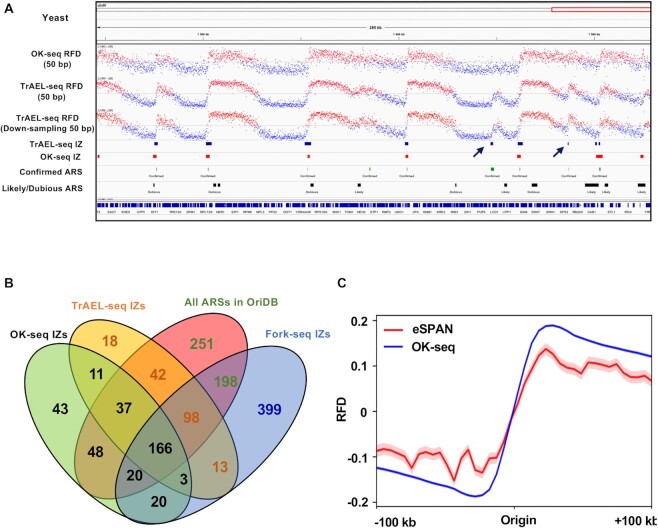
Genome-wide RFD profiles obtained using TrAEL-seq and eSPAN datasets. (**A**) RFD profiles and the corresponding IZs (50 bp bin size) from yeast OK-seq and TrAEL-seq datasets (11). The known yeast origins (ARS) were downloaded from OriDB. Arrows indicate two IZs (chrIV:1447102–1448300, chrIV:1486452–1486950) identified in the TrAEL-seq dataset but not in the OK-seq dataset. (**B**) Venn diagram showing the overlap between OK-seq IZs (*n* = 348), TrAEL-seq IZs (*n* = 380), FORK-seq initiation events (*n* = 4964), and known origins (ARS) from OriDB (*n* = 829); overlap means that the closest distance between the centre of an IZ and of an ARS is <2 kb. When origins in one dataset overlapped with several origins in the other datasets, only one number was provided with the following priority order: OK-seq > TrAEL-seq > OriDB > FORK-seq. It should be noted that there are more origins unique to FORK-seq because this is a single-molecule technique that allows identifying also initiation events with very low frequency. (**C**) Metagene average RFD profiles computed using the OK-seq and eSPAN datasets from mouse embryonic stem cells (8). The mean and standard error bands are shown only for the eSPAN dataset because the standard error bands for the OK-seq dataset were too narrow to be seen.

Besides OK-seq data, the OKseqHMM toolkit can be used to compute the RFD profiles from sequencing data obtained with other techniques. As a demonstration, our toolkit was tested using previously published eSPAN (8) and TrAEL-seq (11) datasets. The RFD data computed from the yeast TrAEL-seq data were very similar to those obtained using OK-seq data (Figure 7A, *R* = 0.93, *P* < 10^−15^). The RFD profile obtained using TrAEL-seq data was of higher quality with less local noise compared with the OK-seq-based RFD profile. This difference does not seem to be explained by the higher coverage of the TrAEL-seq data (∼2-fold more reads) compared with the available OK-seq data. Indeed, TrAEL-seq data displayed a lower local noise profile also after down-sampling to the same coverage as OK-seq data (Figure 7A). The better RFD profile obtained with TrAEL-seq data facilitated the detection of replication initiation regions in some cases. For example, an IZ was identified in the TrAEL-seq RFD profile at chrIV:1486452–1486950, but not in the OK-seq data due to the higher local noise (Figure 7A). To further evaluate the IZs detected with different techniques, the IZs identified with FORK-seq data (28) also were integrated. FORK-seq is a nanopore sequencing method that allows mapping replication initiation within single DNA molecules. Comparison of the TrAEL-seq IZs, OK-seq IZs, FORK-seq IZs and yeast ARSs showed that 77% (271/348) of OK-seq IZs and up to 89% (339/380) of TrAEL-seq IZs were found within 2 kb from a known ARS. Moreover, 75% (203/271) of OK-seq IZs associated with ARSs were detected by TrAEL-seq, and ∼69% (186/271) of them were found in FORK-seq data (Figure 7B). Notably, a small percentage of initiation sites that were not associated with OriDB origins were robustly detected by analysing OK-seq, TrAEL-seq and FORK-seq data with our toolkit (Figure 7B, Supplementary Figure S5A-C). This supports the previous observation that replication initiation in yeast can also occur at loci barely enriched in ARS consensus sequence motifs, although with low frequency (28). Again, the IZs identified in both OK-seq and TrAEL-seq datasets showed higher firing efficiency and larger size than those identified in only one dataset (Supplementary Figure S5D, E). Finally, OKseqHMM was used to compare OK-seq and eSPAN data (8) of mouse embryonic stem cells (mESC). However, due to the lower amount of reads in the eSPAN dataset, the obtained RFD profiles were too noisy to perform a robust IZ calling, despite using a larger window size (e.g. 10 kb smoothing window instead of 1 kb window). This is explained by the fact that our detection method is read depth-dependent. Nevertheless, the mean RFD profile obtained with the eSPAN dataset was similar to the RFD profile obtained with OK-seq mESC data around the IZs identified in the OK-seq dataset (Figure 7C).

## DISCUSSION

Genome-wide replication fork directionality data help to understand many biological processes, such as TRCs, replication-associated mutagenesis, replication couple epigenetic maintenance. Here, we presented OKseqHMM, a comprehensive R package that analyses OK-seq data from various cell types and species to generate and visualize RFD and OEM profiles at high resolution and genome-wide, as well as to generate the average profiles/heatmaps of the regions/genes of interest. The toolkit also allows accurately detecting replication initiation/termination zones with an HMM algorithm. To our knowledge, this is the first bioinformatics tool to handle and analyse RFD data obtained from sequencing datasets generated using various techniques. The toolkit is based on R, and should be easy to use by bioinformaticians and also biologists (e.g. via Rstudio).

We successfully used OKseqHMM to analyse available OK-seq datasets from different species (yeast and mouse) and also normal and cancer human cell lines (Table 1). This toolkit is an important resource for many research communities with different interests (e.g. DNA replication programs, TRCs, replication-associated chromatin organization, replication-associated mutations, genome instability and cancer genomics). Importantly, besides OK-seq, many new techniques have been developed to study DNA replication and to provide replication fork direction data. These include eSPAN and SCAR-seq to perform stranded sequencing of BrdU- or EdU-labelled nascent replicated DNA associated with specific histone modifications, and also TrAEL-seq and GLOE-seq based on the single-stranded end present on specific replicative templates. Here, we demonstrated that OKseqHMM can be used to analyse also data obtained with these techniques with high-quality results (Figure 7). Notably, label-free techniques (e.g. TrAEL-seq) that require fewer cells to generate high-quality RFD profiles, compared with OK-seq, will provide a good alternative to study DNA replication and genomic instability in different cell types exposed to different stress conditions.

Importantly, a multi-scale method is needed to extract the replication initiation/termination information from human RFD profiles because replication initiation zones have various sizes, from 10 to 100 kb (Figures 4–6, Supplementary Figure S3). Sizes are even larger for replication termination zones, from 120 to 500 kb. In previous studies, without an adapted bioinformatics tool for OK-seq data analysis, scientists often used peak calling methods to the OEM profile at a fixed scale in order to identify replication IZs and/or TZs (8,11,25). However, this method identifies only the IZ/TZ centres, but not the precise boundaries of individual IZ/TZ. We previously described an HMM method to identify the precise location of IZs/TZs from OK-seq data at multiple scales (6); however, the lack of an easy-to-use bioinformatic tool limited its application by other groups. Therefore, we developed the current tool to fill this gap. We combined all necessary analysis steps in two main R-based functions that include (i) calculating RFD profiles genome-wide from sequencing data; (ii) generating OEM profiles at multiple scales to visualize RFD transitions; (iii) replication initiation and termination zone calling based on a 4-stage HMM algorithm; and (iv) analysing RFD/OEM profiles around regions of interest (Figure 1). The obtained genome-wide profiles and segmentation results are outputted as bigwig and bed files, respectively, that can be easily visualized using genomic browsers (e.g. IGV or UCSC genome browser). It should be noted that the initiation parameters, such as the transition and emission probabilities of HMM, are defined based on the OK-seq datasets of human cells. Here, we showed that these parameters are quite robust and can be also applied to OK-seq datasets from yeast (Figure 3) and mouse cells (Figure 7, Table 1) with satisfactory results. To further improve IZ/TZ calling, the transition and emission probability matrix and the threshold of minimum read count per bin, which are the main parameters of the functions, could be easily adjusted by users based on the sequencing depth and data quality of their datasets. As highlighted by the comparison of the results obtained from the two biological replicates of yeast OK-seq data, the RFD profiles are very reproducible; however, we strongly recommend to use biological replicates to reduce the potential false detection of IZ calling due to the local variation in the RFD profiles (Supplementary Figures S1 and S2).

One advantage of our OKseqHMM method is that in addition to the replication initiation and termination states, we also included two flat states that allow identifying large domains with high fork polarity, call high-RFD regions (i.e. regions replicated by long-travelling unidirectional replication forks poor in replication initiation zones), an important information, for example, to study common fragile sites (31). In the future, besides HMM, we may include other methods and algorithms in our toolkit, such as (i) a combination of peak calling, for instance MACS2 (32), and multiple-scale OEM profiling; (ii) identification of structural changes in linear regression models from a generalized fluctuation test framework (e.g. the ‘strucchange’ R package) (33) using the RFD profiles; or (iii) a multi-scale analysis of RFD profiles using a wavelet-based signal-processing algorithm (34). Moreover, a comparison between biological replicates and/or between OK-seq datasets obtained with different techniques showed that we missed some bona fide IZs, especially those with lower firing efficiency because we used a strict strategy to reduce false positive detection of IZs (Supplementary Figures S2, S3 and S5). It would be interesting to improve the capacity to discriminate between IZs with low firing efficiency and background noise to reduce false negative results.

In the future, with technical improvements, we might be able to extend the OKseqHMM toolkit to study the extrinsic (cell-to-cell) or intrinsic (homolog-to-homolog) variability of DNA replication, if we can obtain data at the single-cell level and/or in an allele-specific manner, as recently achieved for replication timing (35,36). Moreover, although conflicts between DNA replication and transcription (TRC) under replicative stress can affect genomic stability and promote cancer development (1), the direct study of TRCs at the genome-wide scale is still challenging. Interestingly, transcription-replication immunoprecipitation on nascent DNA sequencing (TRIPn-seq) has been recently developed to try to fill this gap (37), although its current labelling length (30 min of BrdU labelling, therefore, ∼50 kb nascent DNA considering that the replication fork speed is 1–3 kb/min) is still too long to directly measure TRCs. TRCs could be investigated by combining TRIPn-seq with our toolkit that provides precise RFD profiles and accurate genomic coordinates of replication origin prediction and TRC locations.

## CODE AVAILABILITY

The bioinformatics tool and all processing data underlying this article are available in the GitHub repository (https://github.com/CL-CHEN-Lab/OK-Seq) and on Zenodo (https://doi.org/10.5281/zenodo.7428883).

## DATA AVAILABILITY

The genome assemblies sacCer3 (yeast), mm10 (mouse) and hg19 (human) were used in the analysis. The raw sequencing data of OK-seq are available with the corresponding accession numbers indicated in Table 1. The OK-seq data of HeLa S3 cells have been deposited at Gene Expression Omnibus (GEO) under accession number GSE193547. The known yeast origins (ARSs) were downloaded from the OriDB website (http://cerevisiae.oridb.org/) (29). The initiation events identified by FORK-seq were obtained from (https://www.biologie.ens.fr/∼hennion/forkseq.html) (28). The replication timing data of HeLa S3 cells generated by Repli-seq (38), i.e. the S50 values (the fraction of S phase at which 50% of the DNA is replicated in a defined genome region) were obtained from (39). The RNA-seq and GRO-seq data of HeLa cells are from (22) and (40), respectively.

## Supplementary Material

gkac1239_Supplemental_File

## Notes

Present address: Xia Wu, Zhongshan School of Medicine, Sun Yat-sen University, Guangzhou, Guangdong 510080, China.
