# Supplementary material for: OKseqHMM: a genome-wide replication fork directionality analysis toolkit
Source: Nucleic Acids Res. 2023 Jan 11;51(4):e22. doi: 10.1093/nar/gkac1239 (PMC9976876; doi:10.1093/nar/gkac1239)

Supplementary Figure S1. Comparison of the results by OKseqHMM in two yeast OK-seq replicates. (A) The merged, replicate 1 (rep1), and replicate 2 (rep2) RFD profiles were calculated at the 50 bp resolution and the corresponding IZs were identified by OKseqHMM. For the merged OK-seq IZs, only IZs robustly detected in both replicates were kept, and the merged OK-seq IZs present in each replicate are shown below the corresponding RFD profile. The confirmed and possible (likely/dubious) yeast ARS were from OriDB. **(B)** Cumulative density distribution based on the closest distances between confirmed ARS and the merged OK-seq IZs (black), and between confirmed ARS and the random simulation control (red). **(C)** Venn diagram showing the number of confirmed origins from OriDB that overlapped with the IZs detected in the two OK-seq yeast replicates; overlap means that the closest distance between the centres of ARS and IZ is <2 kb.

Supplementary Figure S2. Comparison of the RFD profiles for IZs shared between two yeast OK-seq replicates and for replicate-specific IZs. (A) Average RFD profiles computed using replicate1 (rep1, red), replicate2 (rep2, blue), and merged (black) OK-seq data for the IZs that overlapped with OriDB confirmed origins (ARSs) in both replicates (left), only in rep1 (middle), or only in rep2 (right). **(B)** Average RFD profiles computed using rep1, rep2, and merged OK-seq data for the IZs that did not overlap with confirmed origins: IZs detected in both replicates (left), only in rep1 (middle), and only rep2 (right). **(C)** Average RFD profiles computed using rep1, rep2 and merged OK-seq data for the OriDB confirmed origins that did not overlap with OK-seq IZs. **(D-F)** Boxplot shows the firing efficiency (D), size (E), and confidence probability (F) of IZs in the indicated subsets (IZs shared or not between replicates and overlapping or not with confirmed OriDB origins).

Supplementary Figure S3. Comparison of OK-seq IZs shared between two HeLa cell lines and cell-line specific IZs. (A) Average RFD profiles for IZs shared between the HeLa S3 (red) and HeLa MRL2 (blue) cell datasets, and for IZs specific to one dataset. **(B)** The same as in (A) for early IZs (S50 <0.4). **(C-E)** Boxplots showing the firing efficiency (C), size (D), and confidence probability (E) of HeLa S3 IZs shared between datasets and of specific IZs (all IZs and early IZs).

Supplementary Figure S4. Genome-wide comparison of OK-seq data from different human cell lines. (A) Pairwise Pearson correlations of OEM profiles obtained using OK-seq data (100 kb) from the indicated human cell lines. **(B)** Pairwise Pearson correlations of the IZs detected in different human cell lines: all detected IZs (left triangle) and IZs located in the constant early regions (right triangle) defined in (30). For each pair of comparisons, the percentage of shared IZs was calculated for each cell type and the mean value of the two was used to draw the comparison matrix.

Supplementary Figure S5. Comparison of IZs detected using OK-seq and TrAEL-seq data. (A) Average RFD profiles of OK-seq (red) and TrAEL-seq (blue) for IZs shared between datasets and for IZs specific to one dataset (all overlapping with OriDB origins). **(B)** Same as in (A) for IZs that do not overlap with OriDB origins. **(C)** An example of bona fide IZ that was identified using both OK-seq and TrAEL-seq data and that was not associated with an OriDB origin. **(D-E)** Boxplots showing the firing efficiency (D) and the size (E) of IZs in the indicated subsets (IZs detected with one or both techniques and overlapping or not with OriDB origins).

Supplementary Figure S1

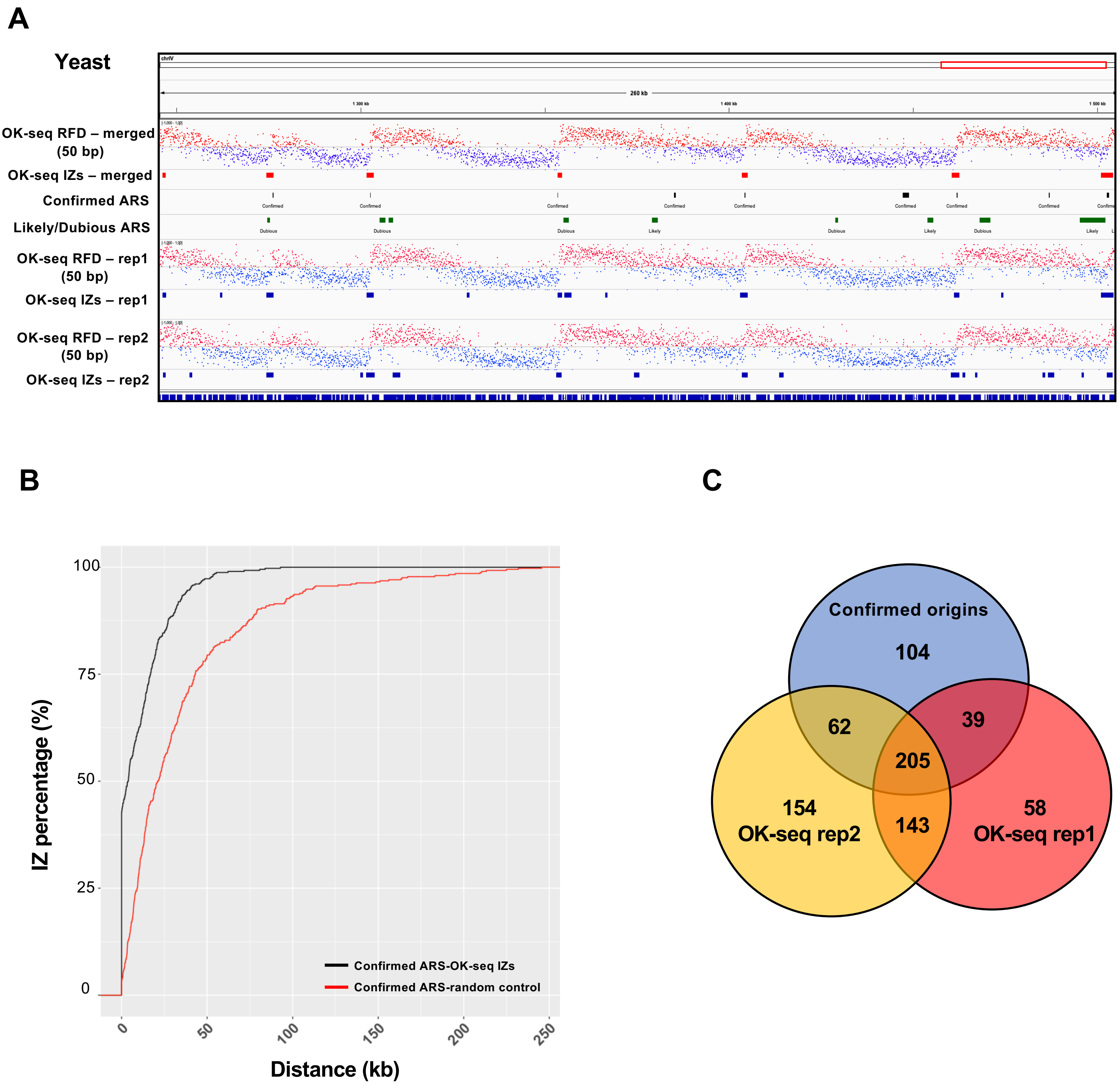

Supplementary Figure S2

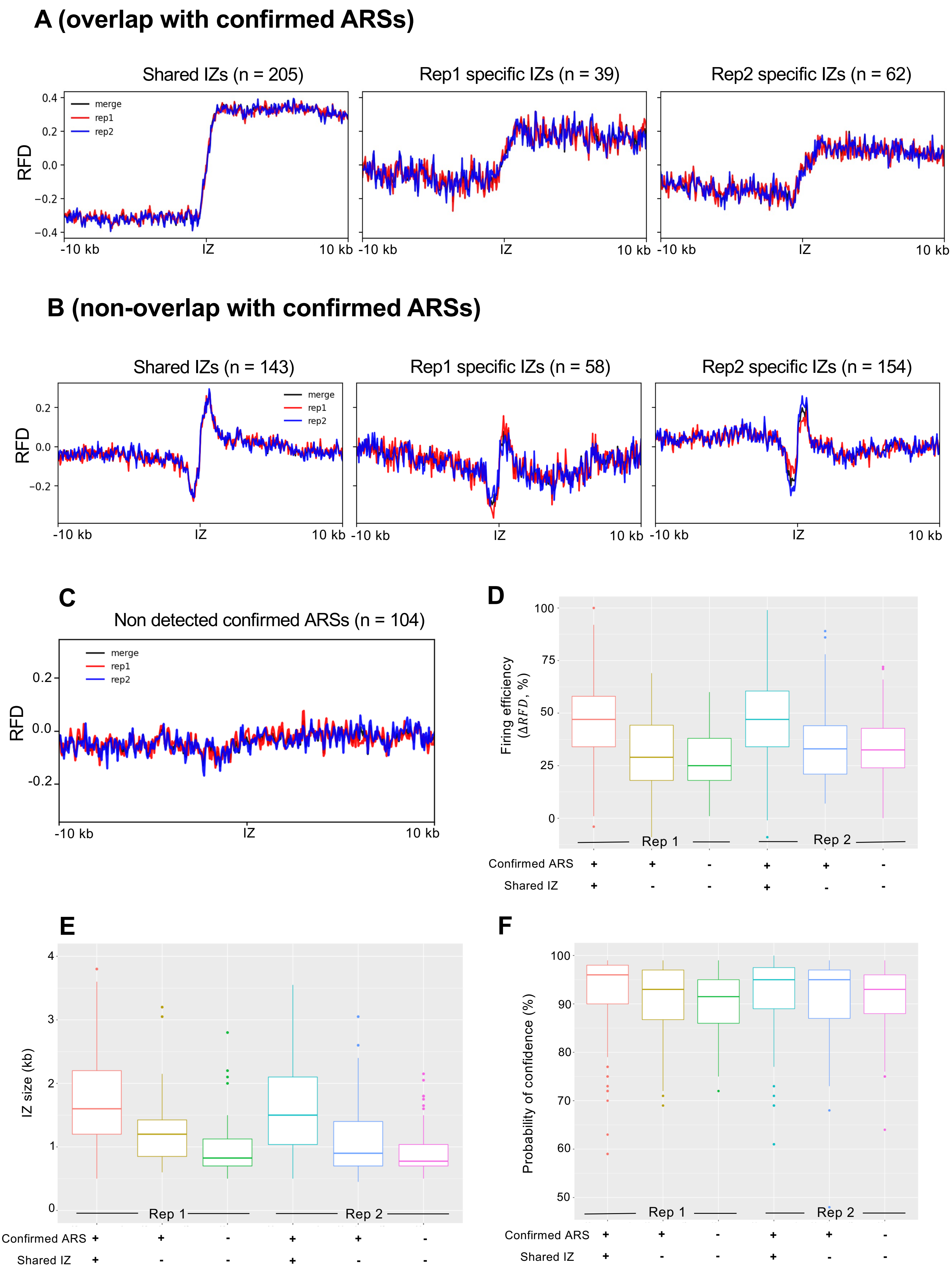

Supplementary Figure S3

A (All IZs)

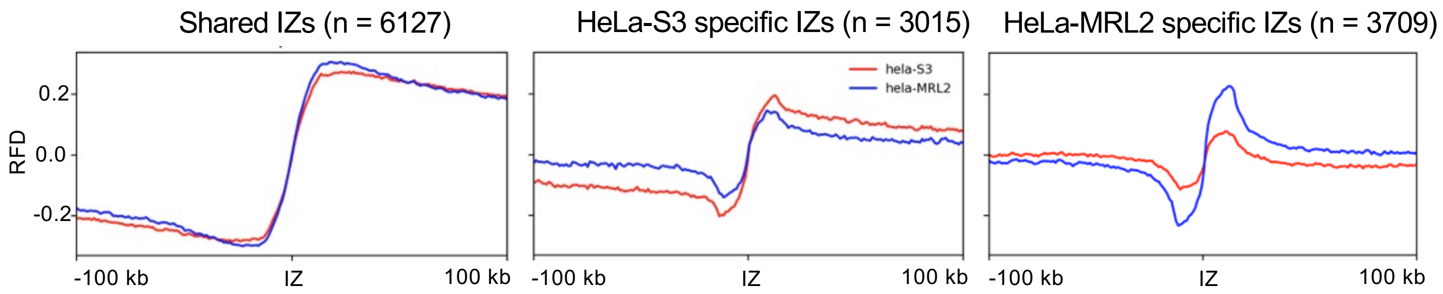

B (Early IZs)

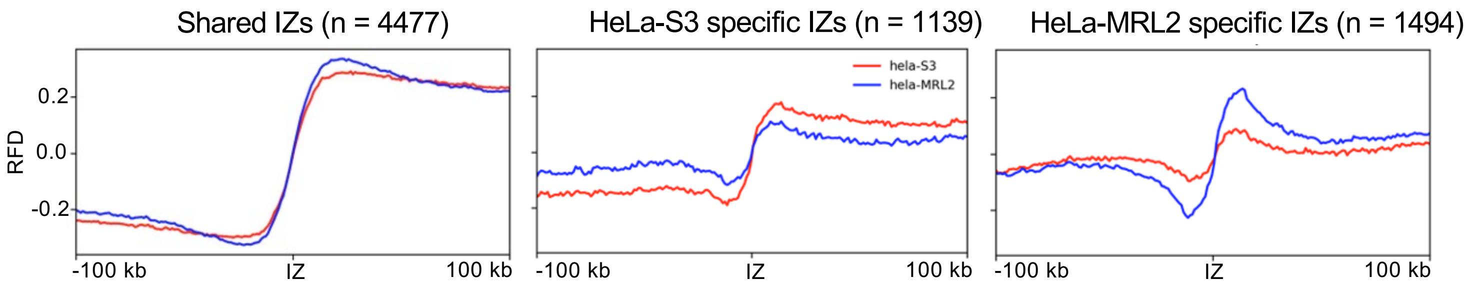

C

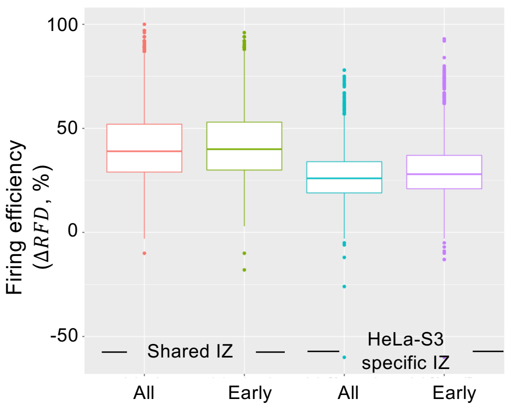

D

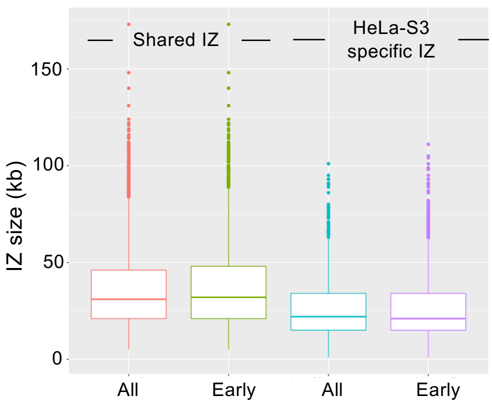

E

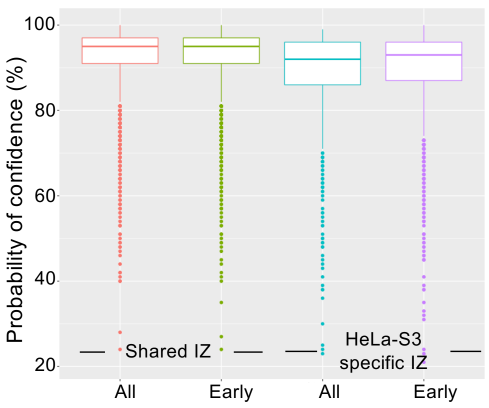

Supplementary Figure S4

A

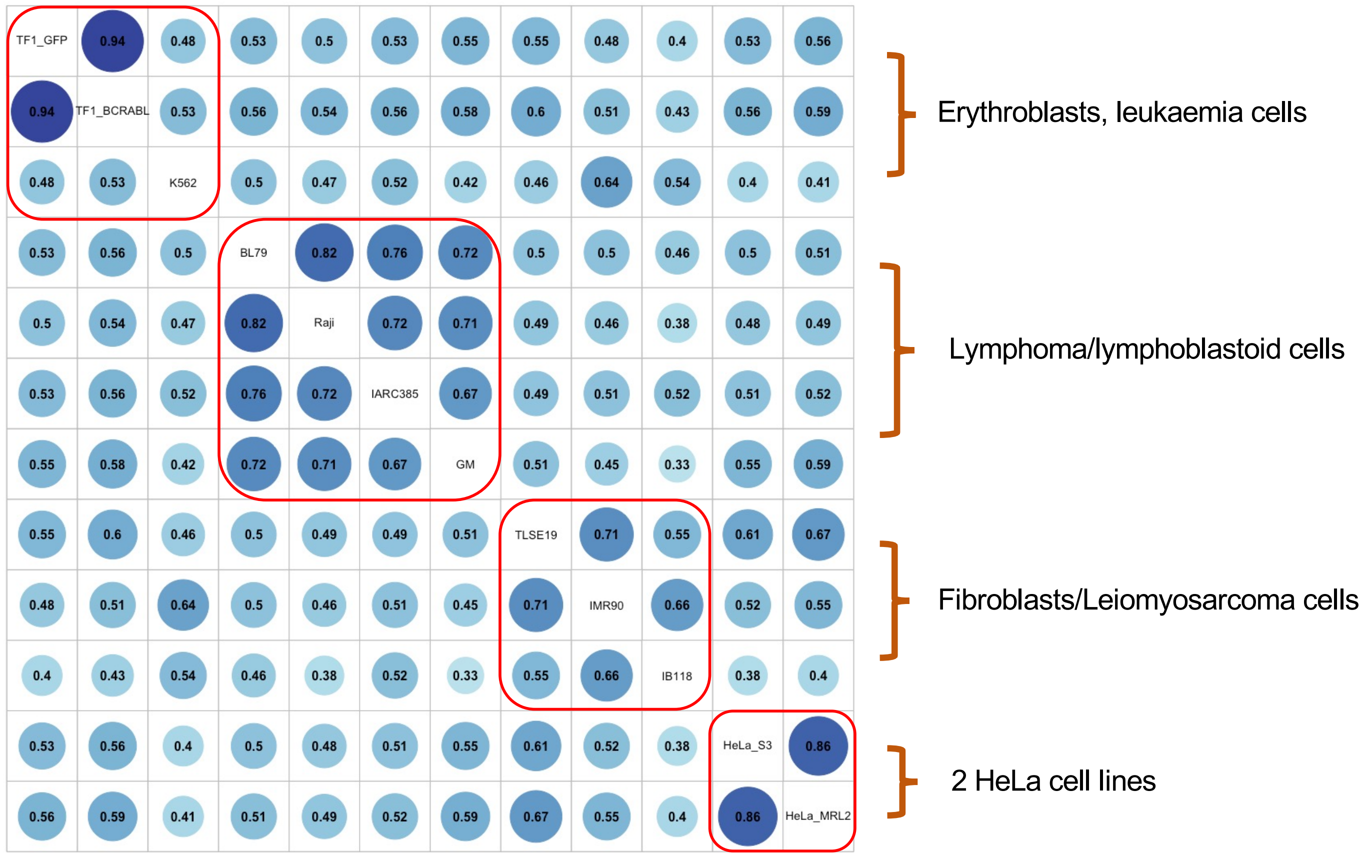

B

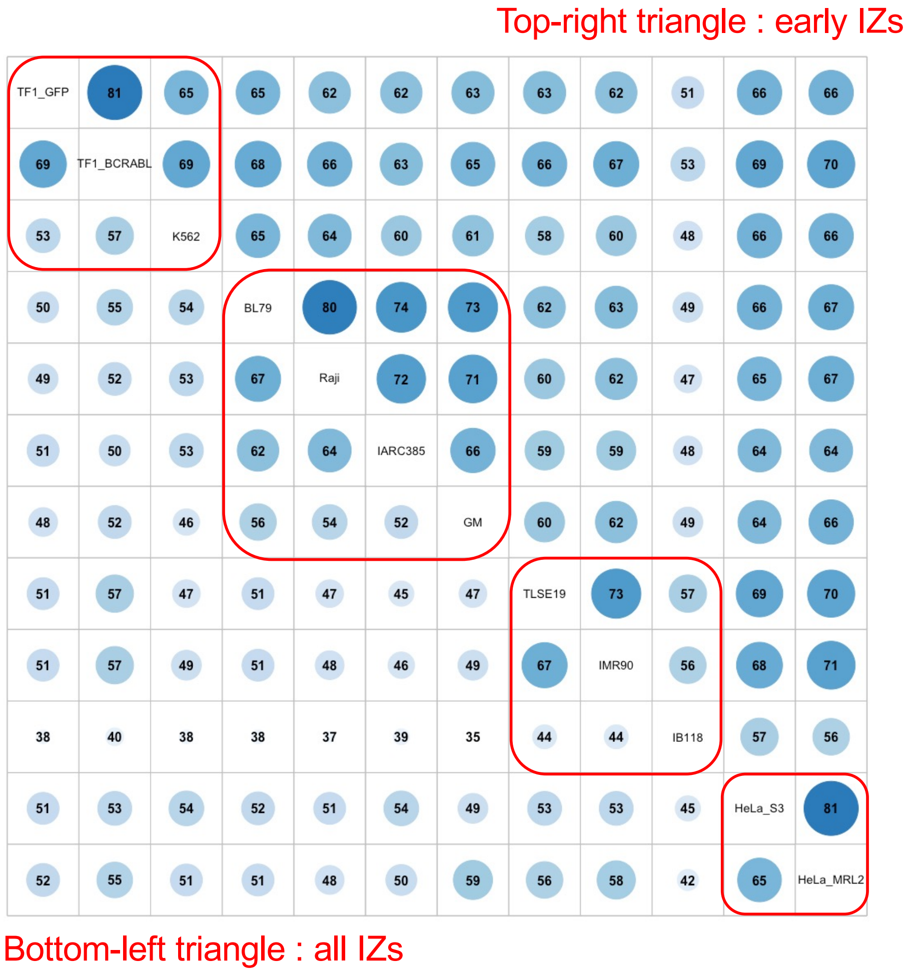

Supplementary Figure S5

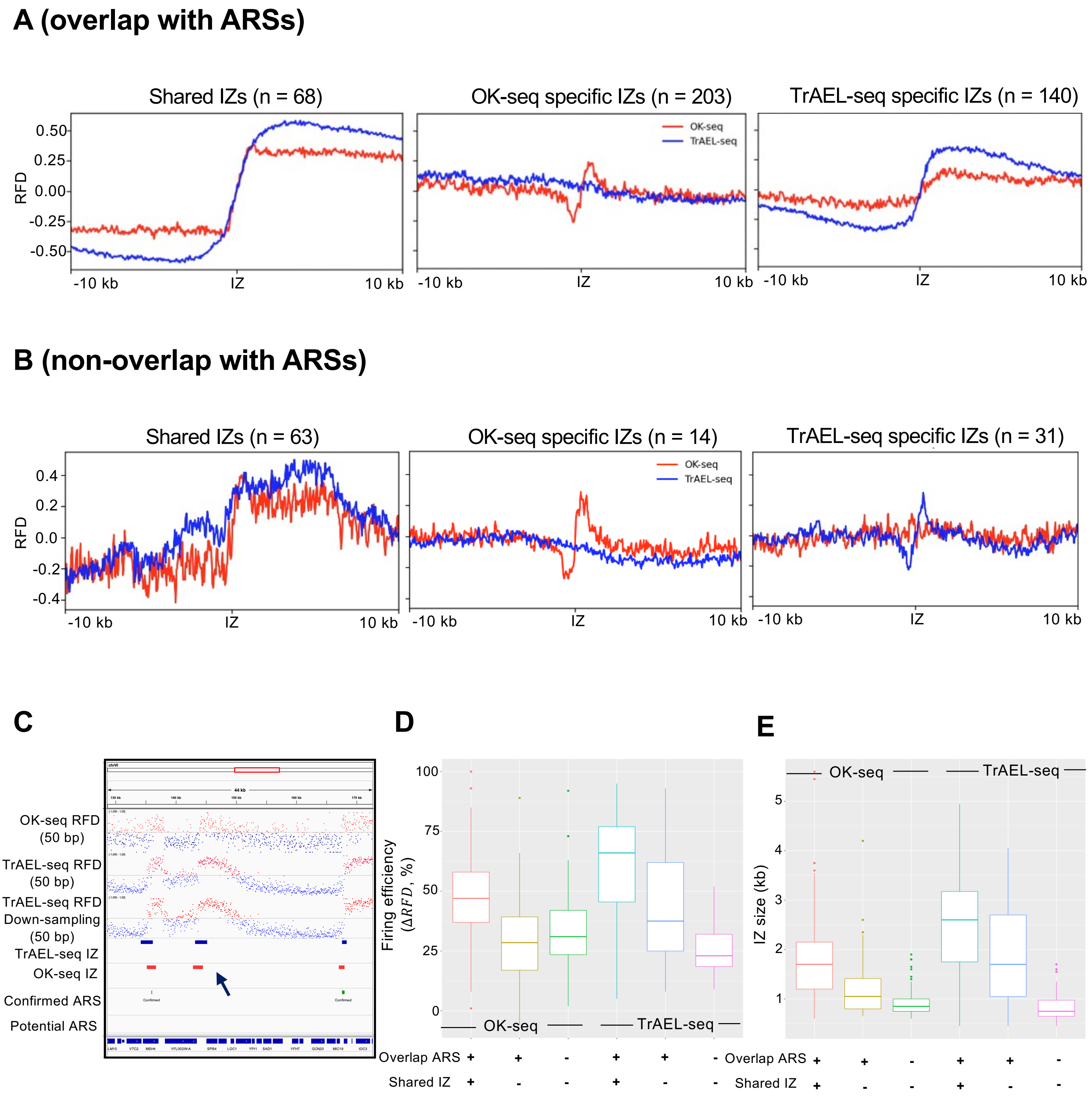

Supplement: gkac1239_Supplemental_File [file gkac1239_supplemental_file.pdf]
